# Gene-set enrichment analysis and visualization on the web using EnrichmentMap:RNASeq

**DOI:** 10.1093/bioadv/vbaf178

**Published:** 2025-07-24

**Authors:** Max Franz, Christian T Lopes, Mike Kucera, Veronique Voisin, Ruth Isserlin, Gary D Bader

**Affiliations:** The Donnelly Centre, University of Toronto, Toronto, ON M5S 3E1, Canada; The Donnelly Centre, University of Toronto, Toronto, ON M5S 3E1, Canada; The Donnelly Centre, University of Toronto, Toronto, ON M5S 3E1, Canada; Princess Margaret Cancer Centre, University Health Network, Toronto, ON M5G 2M9, Canada; The Donnelly Centre, University of Toronto, Toronto, ON M5S 3E1, Canada; The Donnelly Centre, University of Toronto, Toronto, ON M5S 3E1, Canada; Princess Margaret Cancer Centre, University Health Network, Toronto, ON M5G 2M9, Canada; Department of Molecular Genetics, University of Toronto, Toronto, ON M5S 1A8, Canada; Department of Computer Science, University of Toronto, Toronto, ON M5S 3G4, Canada; The Lunenfeld-Tanenbaum Research Institute, Sinai Health System, Toronto, ON M5T 1V4, Canada

## Abstract

**Summary:**

EnrichmentMap: RNASeq (enrichmentmap.org) is an intuitive, web-based app for gene-set enrichment analysis and visualization, specifically supporting two-case RNA-Seq experiments for Homo sapiens. The web app introduces a simplified user interface, faster processing times, and eliminates the need for software installation compared to running similar workflows in the Cytoscape desktop software, catering to biologists with minimal computational experience. EnrichmentMap: RNASeq is a new type of Cytoscape web app that is interoperable with Cytoscape.

**Availability and implementation:**

The app is available to use at enrichmentmap.org and the source code is available at github.com/cytoscape/enrichment-map-webapp.

## 1 Introduction

Gene-set enrichment analysis (GSEA) (Subramanian *et al.* 2005) is important for the interpretation of large gene lists from high-throughput genomic experiments. EnrichmentMap (Merico *et al.* 2010), a network-based visualization tool, has facilitated this process by organizing GSEA (Subramanian *et al.* 2005), g:Profiler (Reimand *et al.* 2007), DAVID (Dennis *et al.* 2003), BINGO (Maere *et al.* 2005), GREAT (McLean *et al.* 2010), and Enrichr (Kuleshov *et al.* 2016) results into interpretable networks. The desktop version of EnrichmentMap, a popular Cytoscape app, requires the installation of multiple components, including Java-GSEA, Cytoscape, and the EnrichmentMap app collection—comprising EnrichmentMap, AutoAnnotate, clusterMaker2, and WordCloud apps (Merico *et al.* 2010, Morris *et al.* 2011, Oesper *et al.* 2011, Kucera *et al.* 2016). While powerful and feature-rich, this setup can be time-consuming to install, learn, and use. Moreover, Java-GSEA, a critical component, can require extended processing times (e.g. 5–20 min) for analysis. There are many web-based pathway analysis tools, such as g: Profiler, Enrichr, Metascape (Zhou *et al.* 2019), PANTHER (Thomas *et al.* 2022), DAVID (Dennis *et al.* 2003), and Reactome (Jassal *et al.* 2020). These tools make enrichment analysis more convenient, but most other tools focus on list-based or tabular outputs, which, while informative, may not provide the same level of intuitive interaction or the ability to explore pathway overlaps and clusters visually as provided by the enrichment map visualization.

EnrichmentMap: RNASeq addresses these issues by providing a web-based implementation of a streamlined enrichment map pathway enrichment analysis workflow. This version is designed for biologists who prefer quick web-based access to a common use case rather than a feature-rich desktop software experience that requires more learning investment. EnrichmentMap: RNASeq focuses on core functionalities such as generating network figures where nodes represent pathways, edges connect pathways by pathway gene set similarity and clusters denote groups of highly overlapping pathways. Unlike the desktop version, which includes advanced features like running GSEA locally, custom clustering with AutoAnnotate, integration with clusterMaker2 for advanced clustering techniques, and WordCloud for customized textual data visualization, the web version is tailored for simplicity and ease of use. It includes automatic clustering with bubble visualization, collaboration capabilities (e.g. web-based sharing of results), and one-step, publication-ready figure export. Further, the web version does not require software to be installed or run locally; everything is accessed through a web browser. Typically, results are generated in under a minute, significantly improving over traditional GSEA and desktop EnrichmentMap applications, which may take 20 times as long (github.com/BaderLab/GSEA_vs_fGSEA, baderlab.github.io/GSEA_vs_fGSEA). EnrichmentMap: RNASeq combines the accessibility of a web tool with the powerful network-based analysis typically found in desktop applications, providing a balance of usability and advanced visualization for pathway analysis.

## 2 Implementation

EnrichmentMap: RNASeq is built using modern web technologies to deliver high performance and usability. The app is open source (MIT license), available on GitHub (github.com/cytoscape/enrichment-map-webapp). Its implementation is based on the EnrichmentMap Protocol (Isserlin *et al.* 2018, Reimand *et al.* 2019). The gene set database used is the Bader Lab gene set database (baderlab.org/GeneSets), with version Human_GOBP_AllPathways_noPFOCR_no_GO_iea_May_01_2024_symbol.gmt.

The server backend is implemented with Node.js and Express.js, providing a scalable and robust foundation for the app. The frontend uses React, to implement a dynamic and responsive user experience, and Cytoscape.js for network visualization. Analysis is implemented by two microservices, derived from the Cytoscape desktop EnrichmentMap app and fGSEA, to provide faster analysis compared to traditional GSEA as used with EnrichmentMap in the Cytoscape desktop app (Subramanian *et al.* 2005, Merico *et al.* 2010, Korotkevich *et al.* 2016). fGSEA creates comparable results to GSEA (baderlab.github.io/GSEA_vs_fGSEA/compare-outputs-of-fgsea-and-gsea.html#compare-nes-values). Whereas GSEA takes on the order of minutes (baderlab.github.io/GSEA_vs_fGSEA/run-gsea-from-within-r.html#timing), fGSEA takes on the order of seconds (baderlab.github.io/GSEA_vs_fGSEA/run-fgsea-from-within-r.html#timing-1). The source code for GSEA and fGSEA comparisons can be found on GitHub (github.com/BaderLab/GSEA_vs_fGSEA). The microservices architecture enables independent development, deployment, and scaling of components, supporting performance, code reusability, and maintainability.

Low-count genes are filtered using the edgeR (Robinson *et al.* 2010) filterByExpr function. edgeR is used over alternatives like DESeq (Anders and Huber 2010), limma/voom (Law *et al.* 2014, Ritchie *et al.* 2015), and Cufflinks (Trapnell *et al.* 2012) because edgeR offers comparatively higher speed results (github.com/BaderLab/GSEA_vs_fGSEA) and to give consistency with the Nature Protocols article for EnrichmentMap (Reimand *et al.* 2019), which uses edgeR in its examples. Other custom processing steps, like batch correction and removing sample outliers are not supported by the app and must be addressed by the user for their data using, e.g. an R script to create a custom gene rank (RNK) file for input into EnrichmentMap: RNASeq.

EnrichmentMap: RNASeq design was guided by an iterative user-centered design process (Norman and Draper 1986). We conducted user interviews with a diverse group of potential users, ranging from wet-lab biologists with minimal computational experience to experienced computational biologists. These interviews helped us understand the varied needs and preferences of our target audience. For instance, the layout of the figure was improved to be more in line with how users would edit their figures for publication, with up-regulated pathways on one side and down-regulated pathways on the other side. Based on the feedback gathered, we refined the interface and workflows to ensure that the tool is intuitive, accessible, and practical for users. This iterative process was important for creating a web application that balances simplicity with the power required for effective gene-set enrichment analysis.

## 3 Features

**Streamlined workflow:** EnrichmentMap: RNASeq provides a streamlined web-based pathway enrichment analysis workflow optimized for RNA-Seq data, focusing on a common use case: differential expression analysis between two conditions in Homo sapiens. The workflow itself is based on the EnrichmentMap Protocol (Reimand *et al.* 2019). This workflow is designed to be user-friendly, with predefined parameters that enable users to achieve high-quality results with minimal manual input and minimal processing time. EnrichmentMap: RNASeq delivers results much faster than the desktop GSEA application traditionally used to make Enrichment Maps. A typical GSEA analysis in this workflow may take 5–20 min while fGSEA, a fast implementation of GSEA (Korotkevich *et al.* 2016), generally takes <1 min (github.com/BaderLab/GSEA_vs_fGSEA).

**RNA-Seq focus**: The application is tailored for RNA-Seq data, which is one of the most prevalent types of high-throughput sequencing data in biological research. This focus allows the platform to provide an efficient workflow for analyzing differential gene expression.

**Input data:** Users must upload one of the following files to initiate their analysis:

- Expression Data File (TSV, CSV, or Excel): This file contains normalized RNA counts, with rows representing genes (HGNC gene names or Ensembl IDs) and columns representing samples (one column per sample). When an expression file is provided, the user specifies their experiment’s classes in the user interface.
- Rank file (RNK): This file, originally defined by GSEA (Subramanian *et al.* 2005), contains a ranked list of genes based on their differential expression between the two conditions. Each row includes a gene identifier and its associated rank, which is inversely correlated with the differential expression *P*-value. Rank files can be generated with software such as edgeR (Robinson *et al.* 2010) as described in (Reimand *et al.* 2019).

**Figure 1.**
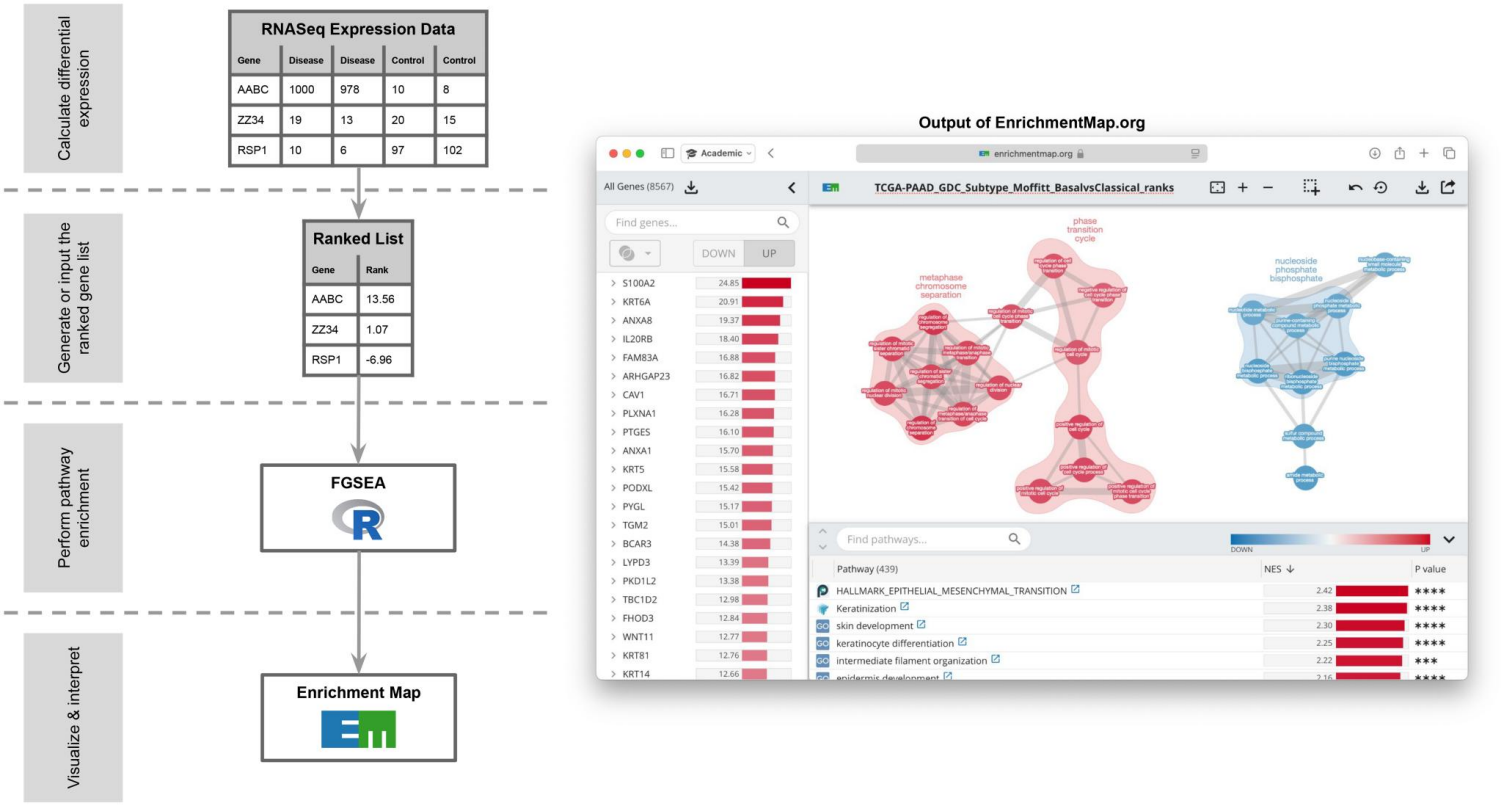
The data flow and user interface of EnrichmentMap: RNASeq.

Examples of each file type are linked from the user interface component requesting the given file (Fig. 1).

**Automatic clustering and visualization:** The application automatically clusters pathways based on gene overlap and presents these clusters using bubble sets visualization (Collins *et al.* 2009). This feature simplifies the interpretation of complex gene-set enrichment results by grouping related pathways together.

**Preconfigured parameters:** To ensure ease of use, EnrichmentMap: RNASeq comes with several pre-set parameters that are tailored for typical RNA-Seq data:

- Normalization: The platform uses the Trimmed Mean of M-values (TMM) method for normalizing RNA-Seq data from edgeR version 4.4.1, which considers variation in sequencing depth across samples (Robinson *et al.* 2010).
- Differential Expression Analysis: Genes are ranked based on *P*-values derived from statistical tests for differential expression between two conditions via edgeR version 4.4.1 (Robinson *et al.* 2010).
- Pathway Enrichment: The application uses the fGSEA (Fast Gene-Set Enrichment Analysis) algorithm version 1.32.2, which is a fast implementation of GSEA, enabling rapid identification of enriched pathways (Korotkevich *et al.* 2016).

The default parameters chosen for EnrichmentMap: RNASeq reflect common practices in RNA-Seq analysis and protocols, ensuring that users can achieve accurate and reproducible results without needing to manually adjust complex settings (Chen *et al.* 2008, Robinson *et al.* 2010, Merico *et al.* 2010). The focus on RNA-Seq as the primary data type simplifies the user experience, catering to the most common experimental design in molecular biology research.

Gene-sets with *q*-value (*P*adj) >0.0001 are removed from the network. Similarity is calculated using the Jaccard method and must have a value of at least 0.5. The Jaccard coefficient is computed as the size of the intersection divided by the size of the union. These values are commonly used, e.g. in the EnrichmentMap Protocol (Reimand *et al.* 2019).

**User interface:** EnrichmentMap: RNASeq’s interface is intuitive and user-friendly for users with varying levels of computational expertise. The responsive design ensures compatibility with various devices, allowing users to access the tool on computers, tablets, and smartphones. The simplified interface focuses on core functionalities, making gene-set enrichment analysis accessible by reducing the computational knowledge needed to execute the workflow.

**Data import:** Users can upload expression data spreadsheets (CSV, TSV, or Excel XLSX) directly from their experiments, streamlining the analysis setup process. Pre-ranked gene lists are also supported for users who have preprocessed their data and ranked their genes with external tools.

**Data export:** Export options include:

- Network PDF image (vector image, infinite DPI) using the PDF export extension (github.com/cytoscape/cytoscape.js-pdf-export)
- SVG image of the NES (normalized enrichment score) color legend
- Network PNG images in various sizes (raster image, 300 DPI)
- Gene-set enrichment analysis results from the fGSEA R package
- Gene ranks
- Gene sets (pathways) corresponding to nodes in the network

These export formats facilitate sharing and publication of results. Further, the result can be exported to Cytoscape for use with the EnrichmentMap desktop app to access more advanced features—such as layout, filters, styles, and other apps. The export archive contains a folder of enrichment data, which can be imported with the EnrichmentMap app within Cytoscape via the “scan a folder for enrichment data” feature.

**Visualization capabilities:** EnrichmentMap: RNASeq provides an interactive visualization, which produces publication-ready figures (i.e. high resolution PNG or vector graphic PDF). Users can manually adjust the network layout by dragging nodes, with color mapping to the NES (normalized enrichment score) for up-regulated and down-regulated pathways. The pathway table allows sorting by NES, *P*-value (BH adjusted *P*-value), and searching by pathway name. Selecting a pathway in the table highlights the corresponding node, and vice versa. The gene list feature supports exploring genes within the entire result or specific pathways, with sorting options and additional information links to NCBI Gene and GeneMANIA databases (Mostafavi *et al.* 2008, Brown *et al.* 2015). Gene lists can be downloaded in TSV format for further analysis.

**Collaboration and sharing:** EnrichmentMap: RNASeq supports collaboration through interactively editable result figures that can be shared via a permalink copied to the clipboard using a Share button. User data is private by default. Others can access a user’s data or results only if the user explicitly shares a result’s permalink. Each enrichment analysis result is stored at unique URLs including a unique identifier of high entropy which is almost impossible for someone to guess. This sort of entropy approach is standardly used to protect easy-to-use, URL-shared documents in online systems, such as Google Docs.

**Use cases:** EnrichmentMap: RNASeq is applicable in various research fields, including cancer research and genetic studies. The application is suited to both bulk RNA-Seq and single-cell RNA-Seq pseudo-bulk data.

**Excluded features:**

- Advanced customization: EnrichmentMap: RNASeq does not include advanced customization options, such as specialized gene expression data processing, manual clustering of gene set nodes, detailed parameter tuning, or integration with additional data types beyond RNA-Seq.
- Extended multi-condition analysis: The application is currently limited to two-case comparisons, meaning it does not support experimental designs with more than two conditions or classes.
- EnrichmentMap: RNASeq supports only Homo sapiens using HGNC and Ensembl gene identifiers.

## 4 Future directions

Future enhancements for EnrichmentMap: RNASeq include support for more organisms and additional cases/classes, support for unranked gene list input, further simplification of the user interface, and integration of community feedback to drive new features and improvements.

As EnrichmentMap: RNASeq continues to evolve, we plan to further integrate it into the Cytoscape ecosystem, which is expanding to the web. For example, we plan to connect EnrichmentMap: RNASeq and Cytoscape Web, an online platform for editing and visualizing network data (Ono *et al.* 2025). This integration would enable users to send their EnrichmentMap: RNASeq results to Cytoscape Web for further refinement and customization. For example, after generating an initial network in EnrichmentMap: RNASeq, users could open the network in Cytoscape Web to apply more sophisticated layout algorithms, add annotations, edit node and edge attributes, or incorporate additional data layers. This would offer a powerful, entirely web-based workflow for both the creation and customization of complex biological networks.
